# Association between maternal risk factors and preterm birth in South Korea: a nationwide cohort study of 795,715 pregnancies

**DOI:** 10.1186/s12884-026-08791-1

**Published:** 2026-02-10

**Authors:** Jaewoo Cha

**Affiliations:** 1 Health Insurance Review & Assessment Service, Wonju, Republic of Korea; 2 Department of Preventive Medicine, Korea University College of Medicine, Seoul, Republic of Korea

**Keywords:** Preterm birth, Adolescent pregnancy, Maternal risk factors, Cohort study, Health disparities, Epidemiology, South korea

## Abstract

**Background:**

Preterm birth (PTB), which is defined as delivery before 37 weeks of gestation, is the leading cause of neonatal morbidity, long-term developmental impairment, and infant mortality. In South Korea, PTB has become a critical concern amid declining fertility, delayed childbearing, and an increased reliance on assisted reproductive technology (ART). Comprehensive population-based evidence of contemporary maternal and healthcare-related risk factors is limited.

**Methods:**

We conducted a retrospective cohort study using Health Insurance Review & Assessment Service claims data for all singleton live births between 2018 and 2022 (*N* = 795,715). Cox proportional hazards model with gestational age as the time axis were used to estimate the adjusted hazard ratios (aHRs) and 95% confidence intervals (CIs). Preterm birth is inherently a time-to-event process, where risk evolves dynamically over gestational age, and key exposures (e.g., pregnancy complications) arise during follow-up. Cox proportional hazards modeling with gestational age as the time axis is therefore epidemiologically justified and widely used in obstetric research. Pregnancy complications were modelled as time-varying covariates, and baseline hazards were stratified according to the delivery facility level.

**Results:**

Significant risk factors of PTB included a history of PTB (aHR 4.05, 95% CI 2.57–6.36), adolescent pregnancy (< 20 years; aHR 2.35, 95% CI 1.26–3.44), severe pregnancy complications (aHR 2.21, 95% CI 2.04–2.38), ART conception (aHR 1.32, 95% CI 1.18–1.47), and a history of miscarriage (aHR 1.34, 95% CI 1.21–1.48). Women covered by Medical Aid, reflecting a lower socioeconomic status, were also at an increased risk (aHR 1.65, 95% CI 1.06–2.57). Although the established model demonstrated excellent discrimination, False labor, while strongly associated with preterm birth, represents a clinically proximate predictive marker rather than an etiological cause, reflecting imminent risk as pregnancy progresses.

**Conclusions:**

This nationwide analysis identified significant associations between recurrent obstetric history, adolescent pregnancy, ART, socioeconomic disadvantage, and the risk of PTB. These findings underscore the importance of early antenatal risk stratification, targeted support for vulnerable populations, and implementation of policies to address the structural determinants of maternal health. Insights from the demographic and healthcare context of Korea may inform global strategies to reduce PTBs.

**Graphical Abstract:**

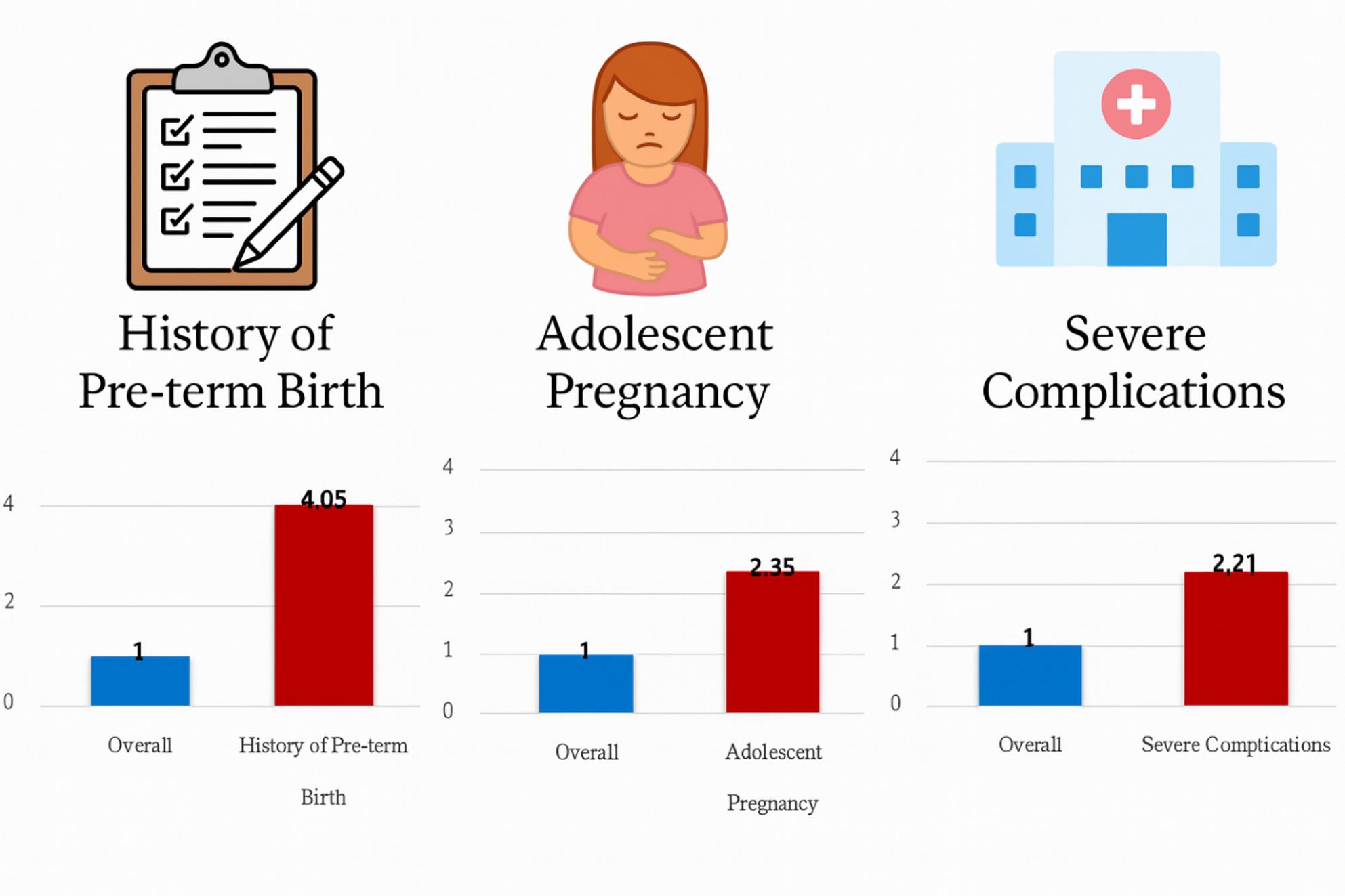

**Supplementary Information:**

The online version contains supplementary material available at 10.1186/s12884-026-08791-1.

## Background

Preterm birth (PTB), which is defined as delivery before 37 weeks of gestation, is the leading cause of neonatal morbidity, long-term neurodevelopmental impairment, and infant mortality worldwide [1, 2]. Each year, approximately 15 million babies are born prematurely, with more than one million deaths attributed to related complications [3]. These outcomes place a substantial burden on families and the healthcare system.

In South Korea, PTB is an increasingly urgent concern amid the demographic crisis characterised by declining fertility rates and delayed childbearing. The number of live births dropped from 326,000 in 2018 to 260,000 in 2021, whereas the total fertility rate fell to a historic low of 0.78 in 2022 [4]. The average maternal age has risen to 33.5 years, with over 35% of births now occurring among women aged 35 or older [5]. This trend is accompanied by the growing use of assisted reproductive technologies (ART), which are associated with a higher risk of complicated pregnancies and preterm deliveries [6–9]. The sustained decline in fertility in South Korea has been accompanied by significant demographic and reproductive changes, most notably delayed parenthood and a growing reliance on ART. The total fertility rate in South Korea remains the lowest among OECD countries, and this demographic trend has driven couples to postpone childbearing into their 30s and 40s. Advanced maternal age is independently associated with adverse obstetric outcomes including hypertensive disorders, gestational diabetes, and an elevated risk of PTB. Delayed fertility increases the demand for ART, which has a unique risk profile. Pregnancies conceived through ART are more likely to involve multiple gestations, underlying subfertility, and intensive medical interventions, factors which have been consistently associated with higher rates of prematurity in both Korean and international cohorts.

It is important to emphasise that declining fertility does not directly cause PTBs. Rather, the fertility decline has reshaped the reproductive landscape, leading to patterns of childbearing and medical interventions that increase the prevalence of known risk factors for preterm delivery. This demographic context underscores the need for continuous population-based studies, not only to monitor temporal changes in the incidence of prematurity but also to disentangle how evolving maternal characteristics (e.g. age, comorbidities, and parity) and healthcare practices (e.g. ART utilisation and obstetric interventions) contribute to PTB risk. Accordingly, the rationale for the present study was to situate individual-level determinants of PTB within the broader context of fertility and demographic transitions in Korea, recognising that the intersection of delayed childbearing, ART use, and increasing obstetric complexity may uniquely shape PTB risk.

Several studies in Korea have investigated the risk factors associated with PTB, including analyses of hospital-based cohorts and earlier claims datasets. These studies identified maternal age, parity, and obstetric complications as important determinants, but were often limited by small sample sizes, single-centre designs, or restricted subpopulations. More recent national database studies have examined specific conditions, such as hypertensive disorders or multiple gestations, but comprehensive analyses of maternal, reproductive, and healthcare-related predictors in the most recent birth cohorts remain scarce. Our study builds on this body of work by analysing approximately 800,000 pregnancies between 2018 and 2022 in a nationally representative dataset, applying robust survival modelling, and situating the findings within the unique demographic context of the low fertility rate and high reliance on ART of Korea.

Although previous studies have identified maternal risk factors such as advanced age, socioeconomic disadvantage, and obstetric history, large-scale population-based studies examining PTBs within the unique healthcare context of Korea remain limited [10, 11]. This is particularly concerning for vulnerable groups, such as adolescents and low-income women, who experience ongoing disparities despite universal health coverage [10]. Emerging evidence highlights additional factors including ART [6–8, 12].

Therefore, this study aimed to use a nationwide claims database of approximately 800,000 live births in South Korea to identify predictors of PTBs and provide risk-stratified care, health policies, and prevention strategies.

## Methods

### Study design

This retrospective cohort study used nationwide administrative claims data. The study period encompassed deliveries between 1 January 2018 and 31 December 2022.

### Study population

The study population included all live infants delivered during the study period. Key maternal exposures were identified, including adolescent pregnancy (defined as age at delivery of < 20 years). These exposures were selected based on their established or suspected relevance to PTB. Additional covariates included maternal age, insurance type (National Health Insurance or Medical Aid), parity, and preexisting medical conditions. These variables were included to control potential confounding influences on pregnancy outcomes. *Supplementary Table 1* summarises the operational definitions and codes of all the variables.

### Data source

Data were obtained from the Health Insurance Review and Assessment Service (HIRA) of South Korea. South Korea operates a universal, single-payer health insurance system, under which more than 99% of deliveries occur in medical institutions and are covered by National Health Insurance or Medical Aid. Consequently, pregnancy- and delivery-related healthcare utilization is comprehensively captured in HIRA claims, providing a robust foundation for population-based research with high external validity.

In South Korea’s single-payer health insurance system, clinical data are entered by licensed healthcare providers at the point of care and submitted as reimbursement claims. All claims are centrally reviewed by the Health Insurance Review and Assessment Service (HIRA) through a standardized adjudication process that includes automated validation, medical expert review, and coding audits prior to reimbursement. Because reimbursement is contingent on accurate diagnostic and procedural coding, this system incentivizes consistency and completeness in clinical documentation. Coupled with universal insurance coverage and the fact that more than 99%(least are not visiting medical institute due to their individual belief) of deliveries occur in insured medical institutions, the HIRA database provides a nationally representative and clinically reliable source for population-based pregnancy research.

Because preterm birth is defined by the timing of delivery along the gestational continuum, gestational age was used as the time axis in Cox proportional hazards models to appropriately capture dynamic risk evolution during pregnancy.

### Exposure or independent variables

We extracted a range of maternal- and pregnancy-related variables from the HIRA data as candidate predictors based on their clinical relevance and prior literature (Supplementary Table 2). These included:

#### Maternal and pregnancy-related variables

Maternal, reproductive, and clinical characteristics were extracted from the HIRA database, based on established associations with preterm births reported in previous studies. The operational definitions and corresponding ICD-10 codes are provided in *Supplementary Table 1*.

#### Maternal demographics

Maternal age at delivery was analysed both continuously and categorically (20–29, 30–34, 35–39, and ≥ 40 years). Nationality (Korean or foreign-born) and insurance eligibility type (employee vs. self-employed/dependent) were also included. Enrolment in the Medical Aid program was used as a proxy for low socioeconomic status. In addition, adolescent pregnancy was defined under 20 years old in the cohort.

#### Reproductive history

Reproductive history included parity (nulliparous or multiparous), previous preterm birth, and a history of miscarriage within five years preceding the index pregnancy. These variables were identified using delivery and diagnosis codes from longitudinal medical records.

#### Assisted reproductive technology

Pregnancies conceived through in vitro fertilisation (IVF) or related procedures were identified from insurance claims within one year before delivery. ART was coded as a binary variable (yes/no) to capture independent risks associated with subfertility and medical interventions.

#### Maternal comorbidities

Major pregnancy-related complications (such as hypertensive disorders, diabetes, placenta previa or placental abruption, and premature rupture of membranes) and pre-existing conditions (such as endometriosis) were included as binary indicators (present or absent). These variables were modelled as time-dependent covariates to reflect their onset during gestation. A summary of these categories is presented in *Supplementary File 1*, and the corresponding ICD-10 codes are also listed in *Supplementary Table 1*.

#### False labor

False Labor was included as an indicator of imminent risk, representing episodes of uterine contractions or cervical changes occurring before 37 weeks of gestation. False labor reflects episodes of abnormal uterine activity or premature cervical ripening occurring before 37 weeks of gestation. Although not meeting criteria for active labor at the time of diagnosis, these events may represent an early warning sign of impending preterm parturition and therefore function as a clinically proximal predictor of preterm birth risk.

### Outcome

#### Preterm birth

The primary outcome was time to PTB, which was defined as delivery before 37 weeks of gestation. Gestational age, derived from claims records, served as the timescale for survival analysis.

PTB were identified with diagnostic codes (ICD-10 code: O60). Only live singleton births were included. Pregnancies reaching 37–42 weeks of gestation were censored at delivery.

Socioeconomic status was assessed based on Medical Aid enrolment, which identifies the lowest-income group. In the Korean single-payer health insurance system, Medical Aid is a government-funded program that covers individuals and households with the lowest income who are exempt from National Health Insurance contributions. Eligibility for Medical Aid is determined by strict income and asset thresholds, and beneficiaries represent the most socioeconomically disadvantaged segment of the population. Accordingly, Medical Aid enrollment has been widely used as a proxy for low socioeconomic status in population-based health research in South Korea.

Hospital accessibility, which considered as a proxy for healthcare service availability and quality, was initially evaluated. For instance, Air Ambulance utilization (for emergency obstetric transfers, equivalent in legal priority to severe trauma cases in South Korea) was explored, as previous studies have reported positive associations with maternal outcomes. We also examined absolute distances (km) from residence to the nearest intensive care unit (ICU), accounting for regional variation. However, these measures were ultimately excluded due to substantial heterogeneity and limited measurement precision; distances and transfer times varied greatly by day, time, traffic flow, and weather conditions, making reliable quantification infeasible.

The analytic cohort was restricted to pregnancies resulting in live births, because gestational timing was incompletely and inconsistently coded for stillbirths in the Korean administrative claims data.

Importantly, history of miscarriage was not excluded; rather, it was treated as the predictive variable. Miscarriage history was ascertained from diagnostic and procedure codes in the five years preceding the index pregnancy. This decision was guided by previous Korean studies using single-payer claims data, which consistently demonstrated that miscarriage was a significant risk factor for subsequent PTBs. Including this factor preserved both the clinical and statistical relevance in the multivariate models, whereas live birth restriction was applied only to the outcome definition and not to exposure.

### Statistical analysis

We applied Cox proportional hazards models to estimate adjusted hazard ratios (aHRs) and 95% confidence intervals (CIs) for the association between maternal and healthcare-related factors and preterm birth (PTB). Given the 6.9% incidence of PTB, odds ratios could overstate associations, whereas hazard ratios more accurately reflect risk over gestational time. Because pregnancy is inherently a finite time-to-event process with variable exposure lengths, this modelling approach reflects its etiological nature and allows incorporation of time-dependent covariates (e.g., gestational complications) that may occur during pregnancy. The gestational age (weeks) was used as the time axis. Pregnancies contributed to person-weeks from delayed entry at the first observed prenatal claim (or at 14 weeks in the sensitivity analysis) to the earliest preterm delivery (< 37 weeks), censoring at 37 weeks, or term delivery (≥ 37 weeks).

Baseline covariates (e.g. maternal age, parity, insurance type, and ART conception) were entered at time zero, whereas obstetric complications with uncertain onset (e.g. gestational hypertension/preeclampsia, placenta previa/abruption, and premature rupture of membranes) were modelled as time-varying covariates that were switched from 0 to 1 at the first recorded diagnosis. To account for structural heterogeneity, the baseline hazard was stratified according to delivery facility level.

Variable selection combines the a priori clinical relevance with data-driven screening. All candidate predictors were first tested in univariate models, and those with *p* < 0.001 (Bonferroni-adjusted) or strong clinical justification were retained in the final multivariate model. The key factors (adolescent pregnancy, parity, and history of miscarriage) are established predictors of PTB, supporting both theoretical and empirical validity. Certain recognised risk factors (e.g. smoking, alcohol use, maternal BMI, and interpregnancy interval) could not be incorporated because of data limitations. Chi-square test are performed first (Supplementary Material 3), and Model diagnostics confirmed the robustness of the model. The proportional hazards assumption was tested using Calibration plots, Bootstrapping(Supplementary Material 4, 5), Discrimination was evaluated using the C-statistic, which reached 0.90, consistent with excellent predictive ability. Multicollinearity was assessed using variance inflation factors (VIF), with all covariates < 10. (Supplementary Fig. 2).

Survival modelling was preferred over binary regression because risk sets evolve dynamically with gestational time and exposure often arises in mid-pregnancy. This approach better reflects obstetric processes and produces results that are directionally consistent with modified Poisson regression estimates.

All analyses were performed using the SAS software (SAS Institute Inc., Cary, NC, USA). Statistical significance was defined as a two-sided *p*< 0.05.

## Results

### Study cohort characteristics

Pregnant women between 2018 and 2022 (*N* = 850,234) were initially identified.

**Fig. 1:**
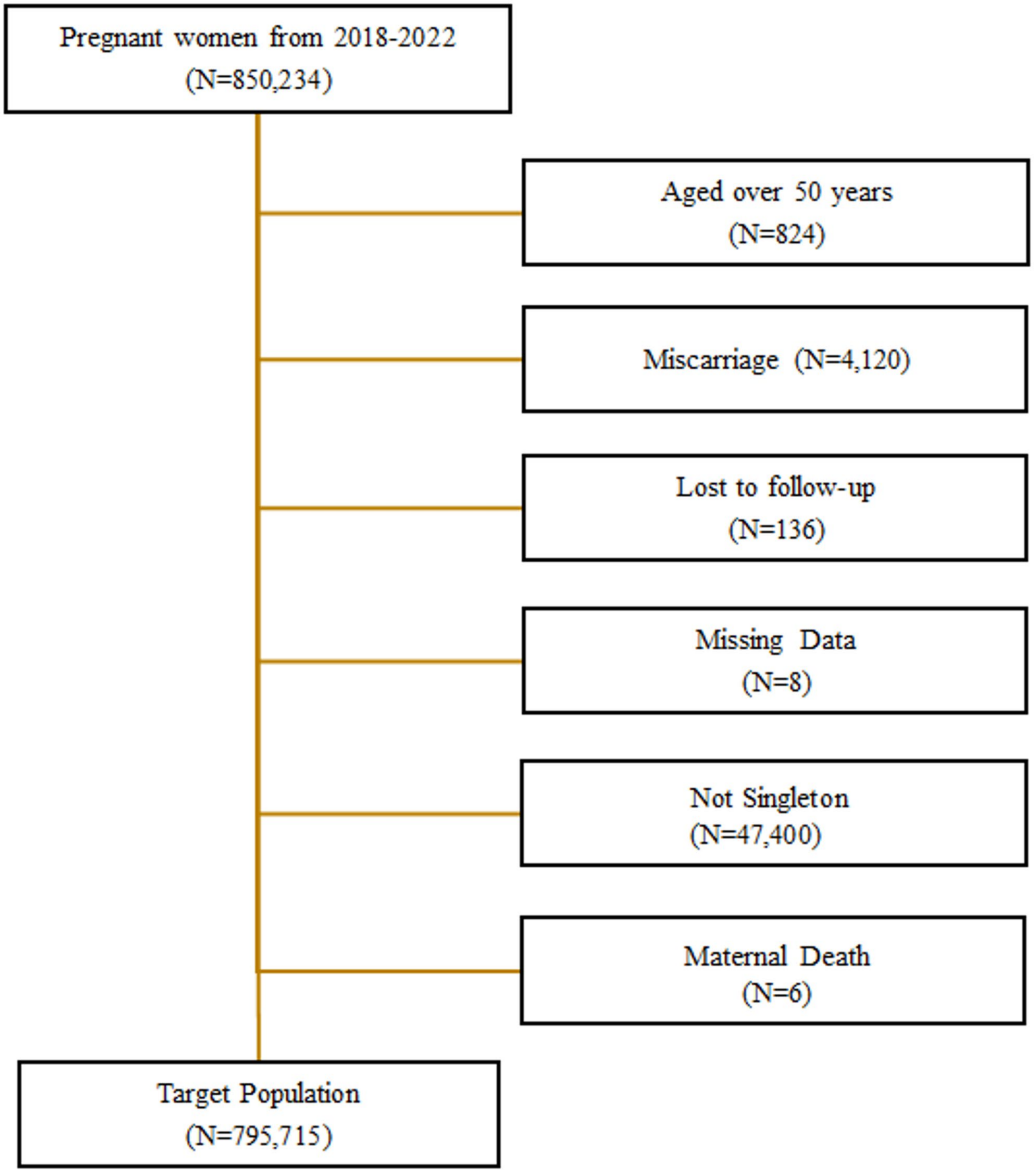
Flowchart of study population selection

In total, 795,715 pregnancies were included in the analysis(Fig. 1). The majority of the participants were aged 30–39 years (68.6%), followed by those aged 20–29 years (27.0%), and a smaller proportion was aged 40–49 years (4.4%). Nearly all individuals were covered by National Health Insurance (99.5%), whereas only 0.5% were Medical Aid recipients.

Nulliparous was present in 48.6% of the patients. Adolescent pregnancies accounted for 2.8% of all cases. A history of miscarriage was reported in 14.2% of the population and 9.3% conceived using ART. 89.9% has experience of false labor, and severe complications occurred in 30.0% of pregnancies. A history of PTB was documented in 0.1% of cases, whereas the observed rate of PTB in current pregnancies was 6.9% (Table 1). Preconception and perinatal risk factors were categorized according to their occurrence timeline (before conception vs. during pregnancy), as detailed in Supplementary File 2.

**Table 1:** General characteristics of the study population

		*N*	%
Total		795,715	100.0
Age			
	20–29	214,843	27.0
	30–39	545,861	68.6
	40–49	35,011	4.4
Insurance type			
	National Health Insurance	791,371	99.5
	Medical Aid Recipient	4344	0.5
Parity			
	Nulliparous	387,257	48.6
	Multiparous	408,458	51.4
Adolescent Pregnancy			
	Yes	22,280	2.8
	No	773,435	97.2
History of miscarriage			
	Yes	113,144	14.2
	No	682,571	85.8
Artificial reproduction Technique			
	Yes	74,015	9.3
	No	721,700	90.7
False Labor			
	Yes	715,388	89.9
	No	80,327	10.1
Severe complications			
	Yes	238,432	30.0
	No	555,661	70.0
History of preterm birth			
	Yes	731	0.1
	No	794,984	99.9
Preterm birth			
	Occurred	54,904	6.9
	None	740,811	93.1

### Cox proportional-hazard model

False Labor was strongly associated with an elevated risk of preterm delivery (aHR 3.87; 95% CI, 2.99–4.75; *p* < 0.001). Severe pregnancy complications also significantly increased the risk (aHR 2.21; 95% CI, 2.04–2.38; *p* < 0.001), aligning with known clinical contributors to early delivery.

A history of PTB remained one of the strongest predictors (aHR 4.05; 95% CI, 2.57–6.36; *p* < 0.001), underscoring its recurrent nature. Use of ART (aHR 1.32; 95% CI, 1.18–1.47; *p* < 0.001) and prior miscarriage (aHR 1.34; 95% CI, 1.21–1.48; *p* < 0.001) were also independently associated with increased risk.

Adolescent pregnancy was significantly associated with PTB (aHR, 2.35; 95% CI, 1.26–3.44; *p* = 0.0011). Insurance type was a significant determinant (*p* < 0.001); Medical Aid recipients had a higher risk of preterm delivery (aHR 1.65; 95% CI, 1.06–2.57) (Table 2). We draw Kaplan-Meier curves for important factors (Supplementary Fig. 1).

**Table 2:** Multivariate Cox proportional hazards regression for risk of preterm birth

		Hazard Ratio (95% CI)	*P*-value
Crude		ref	-
Insurance type			< 0.001
	National Health Insurance	ref	
	Medical Aid recipient	1.65 (1.06–2.57)***	
Parity			0.22
	Nulliparous	0.95 (0.88–1.03)	
	Multiparous	ref	
Adolescent Pregnancy			0.0011
	Yes	2.35 (1.26–3.44)	
	No	ref	
History of miscarriage			< 0.001
	Yes	1.34 (1.21–1.48)***	
	No	ref	
Artificial reproduction technique			< 0.001
	Yes	1.32 (1.18–1.47)***	
	No	ref	
False Labor			< 0.001
	Yes	3.87 (2.99–4.75)***	
	No	ref	
Severe complications			< 0.001
	Yes	2.21 (2.04–2.38)***	
	No	ref	
History of preterm birth			< 0.001
	Yes	4.05 (2.57–6.36)***	
	No	ref	

## Discussion

This study provides important insight into maternal and healthcare-related risk factors for PTBs in South Korea based on a robust, nationally representative cohort of 795,715 pregnancies. Our findings reveal several significant predictors of PTB. A history of PTB has emerged as one of the strongest risk factors, underscoring the importance of obstetric outcomes in anticipating future pregnancy risk. We also observed a strong association between a diagnosis of false labor and subsequent preterm birth. Although false labor should be interpreted as a predictive clinical marker of imminent preterm risk rather than a causal factor, it is typically recorded when women present with uterine contractions or symptoms suggestive of preterm labor that do not initially meet diagnostic criteria for active labor. In this context, the finding likely reflects a clinical harbinger of imminent preterm delivery rather than an independent etiologic risk factor. Women coded with false labor may already be in the early trajectory of preterm parturition, which explains the elevated hazard observed in our time-to-event analysis. This result underscores the importance of careful monitoring and timely management of women presenting with preterm labor–like symptoms, while also highlighting the need to interpret this association as a proximate clinical marker rather than a causal determinant of preterm birth.

Adolescent pregnancy was also significantly associated with preterm delivery, which is consistent with global evidence. Severe pregnancy complications, including hypertension, preeclampsia, and placental abnormalities, markedly increase the risk of PTB. Although adolescent births are rare in South Korea, they constitute a well-recognised vulnerable population, highlighting the importance of targeted interventions. Additionally, The elevated risk observed among Medical Aid recipients indicates that socioeconomic gradients in preterm birth persist despite universal health insurance coverage. Medical Aid status may reflect underlying social and clinical vulnerabilities—such as higher comorbidity prevalence, barriers to timely antenatal care, and chronic psychosocial stress—rather than differences in formal access to maternity services. Although risk factors for PTB such as prior prematurity, adolescent pregnancy, and ART utilisation are well established, their significance must be continually reassessed within specific demographic and health system contexts. South Korea represents a particularly compelling case: it has the lowest fertility rate globally, one of the highest rates of ART use, and rapidly evolving maternal health policies aimed at addressing high-risk pregnancies. These unique conditions alter the distribution of risk factors and reshape the clinical and policy landscapes of prematurity. Therefore, our study contributes new value by quantifying maternal and healthcare-related risk factors in a contemporary nationwide Korean cohort, providing evidence that is directly relevant to the design and evaluation of ongoing interventions to improve maternal and neonatal health.

Although a C-statistic of 0.90 may appear unusually high for preterm birth prediction, this level of discrimination is expected in a gestational-age–based survival model that intentionally incorporates time-varying, clinically proximate markers. Variables such as false labor and severe pregnancy complications do not represent baseline risk factors; rather, they function as dynamic clinical signals that arise close to the event and update risk estimates as new information becomes available during follow-up, thereby substantially improving concordance. Accordingly, the high C-statistic reflects the model’s design for ongoing, dynamic risk assessment across gestation, rather than for one-time early pregnancy risk screening (Supplementary Material).

Several previous studies have examined risk factors for PTB in the Korean context. Kim et al. (2005) conducted a multicentre hospital-based cohort study of approximately 2,600 pregnancies and reported associations between vaginal bleeding, low income, prior spontaneous abortion, and history of preterm delivery [13]. However, the study was limited by its relatively small sample size and lack of population-level generalisability. More recently, Ouh et al. (2018) used national insurance data to investigate the recurrence risk and found that women with prior PTB, particularly in the second pregnancy, had a substantially higher risk of recurrence in subsequent pregnancies [14]. Although previous studies were informative, this study focused exclusively on recurrence patterns rather than on broader maternal or healthcare-related factors.

Other investigations have highlighted contextual determinants. Using national birth registry data, Woo et al. (2016) demonstrated seasonal variations in PTBs with elevated risks associated with summer and fall seasons, although their ecological analysis did not address individual-level risk factors [15]. Kim et al. (2023) developed the Korean PTB Risk Assessment Scale (PBRAS-K) for use in high-risk maternity settings, underscoring the importance of early screening and intervention [16]. However, this study used a relatively small clinical sample and did not provide population-based evidence.

Taken together, these studies confirm the relevance of established risk factors in Korea but are constrained by older data, specialised subgroups, or narrow analytic aims. To date, no comprehensive contemporary nationwide assessment has integrated maternal demographics, reproductive history, ART, and healthcare access within the demographic context of declining fertility and high ART utilisation. The present study addresses this gap by analysing nearly 800,000 pregnancies between 2018 and 2022 and provides updated population-based evidence on the maternal and healthcare-related determinants of PTB in South Korea.

Our results align with global findings that identify adolescent pregnancy, previous PTB, and severe maternal complications as strong predictors of preterm delivery [6, 7]. These associations reflect well-established mechanisms, such as biological immaturity, inadequate prenatal care, and psychosocial stress in adolescents, as well as the cumulative obstetric risks associated with hypertensive disorders and placental abnormalities. The observed disparities among socioeconomically disadvantaged groups are also consistent with international evidence on the social determinants of maternal and perinatal health [10, 11]. Moreover, the growing role of ART as a contributor to PTB risk has been noted in other high-income countries, including Japan, Italy, and Europe, where delayed parenthood and ART utilisation are reshaping reproductive health trends [9, 17, 18].

The novelty of our study lies in its methodological and contextual strengths: (1) the robustness of nationwide claims data, ensuring near-complete capture of pregnancies and maternal histories; (2) the ability to distinguish ART-related conceptions within administrative data, a rising and policy-relevant risk factor; (3) the backdrop of Korea’s unprecedentedly low fertility rate and rapidly evolving maternal health policies; and (4) the integration of both clinical and structural determinants of prematurity in a homogeneous, universal-coverage population. Together, these elements distinguish our findings from those of previous Korean and international studies and highlight their direct applicability to contemporary policy interventions. Especially, A central contribution of this study lies in situating established risk factors for preterm birth within South Korea’s unprecedented demographic context. With a total fertility rate of 0.78 in 2022 and ART accounting for 9.3% of pregnancies, reproductive patterns in Korea are increasingly characterized by delayed childbearing, medicalized conception, and a rising concentration of obstetric risk. Our findings suggest that ART use and adolescent pregnancy do not operate in isolation but interact with this broader demographic shift, reshaping the distribution of preterm birth risk and underscoring the need for risk-stratified maternal health policies tailored to a low-fertility, high-ART setting.

These findings underscore the need for early identification and targeted intervention to mitigate adverse outcomes in high-risk groups. For adolescents, youth-oriented health strategies such as comprehensive sexual health education, early pregnancy screening, and integrated social support systems may be more effective than broad population-wide programs [6, 8, 11, 12]. For socioeconomically disadvantaged women, targeted outreach programs and community-based care models could help address persistent disparities in maternal health outcomes.

South Korea introduced several key initiatives to address these challenges. Notably, in 2024, the government will implement tailored support measures for preterm infants, including aligning healthcare benefit periods with the expected date of delivery, expanding financial support for neonatal care, and strengthening maternal–neonatal intensive care networks. Developmental support programs have been scaled to the national level and comprehensive maternal health management packages have been introduced to enhance parental assistance. The Protected Birth and Birth Notification System, launched in July 2024, mandates birth reporting within 14 days and provides an anonymous birth option for mothers in crisis, thereby promoting safe deliveries and reducing maternal and infant mortality risk.

The policy initiatives introduced in Korea can be interpreted in light of the risk profiles identified in this study. Our findings highlight that preterm birth risk is strongly concentrated among women with prior adverse obstetric histories, pregnancy complications, and socioeconomic vulnerability, underscoring the need for targeted financial and healthcare support during pregnancy and the perinatal period. In this context, recent measures such as individualized maternity benefits—including the pregnancy medical allowance (500,000 KRW e-voucher), subsidies for assisted reproductive technology regardless of income, expanded postnatal care for high-risk households, and the First Meeting Voucher (2,000,000 KRW)—may help mitigate economic barriers to timely and adequate maternal care. In addition, amendments to the Mother and Child Health Act in February 2024, which expanded healthcare infrastructure for high-risk pregnancies, strengthened mental health screening and counselling, enhanced support for multiple gestations, and increased breastfeeding facilities, are well aligned with the clinical risk stratification suggested by our results. Collectively, these policies reflect a shift toward risk-responsive maternal health investment in the setting of declining fertility and a growing burden of high-risk pregnancies, and they illustrate how population-based evidence can inform comprehensive maternal health strategies in other high-income countries facing similar demographic challenges.

This study has several strengths. We analysed a comprehensive nationwide cohort using high-quality administrative claims data to ensure strong external validity. Rigorous statistical methods, including Cox proportional hazards modelling, produced excellent predictive performance (C-statistic = 0.90), supporting the robustness of the results. Novel predictors, such as adolescent pregnancy and history of PTB, were evaluated, contributing new insights into under-recognised risk factors. Moreover, we used survival analysis because the risk set dynamically changes with gestational time and pregnancies differ in the observed at-risk duration. Treating PTB as a simple binary outcome disregards time ordering and can inflate or attenuate the effects when exposure occurs during mid-pregnancy. Modelling gestational time with delayed entry and time-varying covariates better reflects obstetric reality and yields results that are directionally consistent with risk ratio estimates from modified Poisson models.

This study also has several limitations that must be acknowledged. Key socioeconomic variables (e.g. employment status and household income) were unavailable, limiting our ability to fully capture the social determinants of health. Because this study was based on retrospective administrative claims data, several social and biological determinants of preterm birth could not be directly assessed. The dataset lacks clinical granularity, including specific indications for preterm delivery or ART. Importantly, we were unable to differentiate between iatrogenic and spontaneous preterm births (SPTBs) despite their distinct etiologies and risk profiles [19]. This limitation is inherent to administrative claims data, in which detailed clinical indications for delivery are not consistently available. As a result, the estimated associations in this study likely reflect a mixture of heterogeneous clinical pathways leading to preterm birth. Nevertheless, the time-varying variables included in our models may differentially capture these pathways. Severe pregnancy complications, such as hypertensive disorders, placental abnormalities, or other maternal-fetal conditions requiring medical intervention, are more likely to reflect pathways related to medically indicated (iatrogenic) preterm birth. In contrast, false labor represents episodes of preterm uterine activity or cervical change occurring prior to active labor and may capture early manifestations of spontaneous preterm parturition.

Accordingly, given the observational nature of this study, the observed hazard ratios should be interpreted as associations rather than causal effects, reflecting distinct clinical trajectories that converge on the shared outcome of preterm delivery. The inability to distinguish between iatrogenic and spontaneous preterm births may therefore attenuate or aggregate pathway-specific associations, and this should be considered when interpreting the magnitude of the estimated effects. Future studies integrating clinical registry data with claims databases will be essential to disentangle these pathways and to provide subtype-specific risk estimates.

Residual confounding due to unmeasured variables cannot be excluded. In particular, important factors such as disease severity, clinical management strategies, medication use, dietary factors, lifestyle-related variables (e.g. smoking, alcohol use, and maternal BMI), detailed sociodemographic characteristics, and the precise timing of clinical measurements were not available in the administrative claims data, which may have influenced the magnitude of the observed associations. Socioeconomic status was approximated using Medical Aid enrolment as a proxy for low income as detailed income or occupation data were not available in the claims database. Although this measure captures economic disadvantages at the population level, it may not reflect the finer gradients of socioeconomic inequality. Their omission may have introduced residual confounding factors, which should be considered when interpreting the observed associations. Another limitation relates to the reproductive history. Although we included miscarriage as a predictor, its role as an antecedent of PTB remains controversial, with conflicting findings in the literature. Some studies have reported an increased risk only for recurrent or late miscarriages, whereas others have reported no significant associations. Therefore, the contribution of miscarriage to our model should be interpreted with caution.

Future research should integrate clinical registries with administrative claims data to enhance clinical granularity, enabling standardized classification and stratified analyses of spontaneous versus iatrogenic preterm births. Such linkage would allow pathway-specific analyses to distinguish medically indicated preterm birth—often associated with severe pregnancy complications such as hypertensive disorders or placental abnormalities—from spontaneous preterm parturition, which may be better captured by clinically proximate markers such as false labor. In particular, nationwide linkage with the National Health Insurance Service (NHIS) database, including annually collected health check-up data, could provide subtype-specific risk estimates and clarify the timing and clinical relevance of time-varying predictors beyond the aggregated hazard ratios observed in claims-based analyses.

In addition, future studies should incorporate more detailed measures of healthcare accessibility, including emergency transport modes and regional capacity indicators, to better capture structural determinants of preterm birth. Nested case–control designs within large population-based cohorts may be particularly useful for estimating odds ratios for accessibility-related factors, thereby complementing survival-based approaches and supporting more targeted, context-sensitive prevention strategies.

Applying advanced machine learning approaches may further strengthen predictive modelling, enabling earlier risk identification and more accurate estimation of intervention effects. Simultaneously, standardised prediction frameworks should be developed to evaluate the impact of preventive medical strategies. Finally, greater attention should be paid to the structural determinants of maternal health, particularly among socioeconomically disadvantaged populations, to ensure that predictive tools and preventive interventions translate into equitable policy designs.

## Conclusion

This large-scale cohort analysis highlighted the need for comprehensive and risk-informed maternal health strategies in South Korea. The primary value of the analysis is service monitoring and quality improvement. Although false labor should be interpreted as a predictive clinical warning sign occurring close to delivery rather than as a causal determinant of preterm birth, policymakers and clinicians should prioritise early antenatal care, risk screening, and continuous support for high-risk groups such as adolescents, women undergoing ART, and those with a history of adverse obstetric outcomes. Proactive identification of these factors can support timely interventions, reduce the incidence of PTBs, and improve maternal and neonatal health outcomes, although these findings are most directly applicable to the Korean healthcare system, which operates under a universal, single-payer insurance structure, and may be most generalizable to settings with similar coverage and access to maternity care.

As global health systems face similar demographic transitions, lessons from the South Korean experience can inform international maternal health strategies and contribute to ongoing efforts to reduce PTBs worldwide [1–3].

## Supplementary Information

Supplementary Material 1. Robinson Classification of the model. Supplementary Figure 1. Kaplan–Meier curves of preterm births according to maternal and clinical factors. Supplementary Figure 2. DAG(Directed Acyclic Graph) Diagram. Supplementary Table 1. General characteristics of factors. Supplementary Table 2. Variable Selections according to comprehensive literature review on related factors. Supplementary File 1. Factors considered in this study (in Detail). Supplementary File 2. Factors According to timeline. Supplementary File 3. Chi-squared test. Supplementary File 4. Calibration Plot. Supplementary File 5. Bootstrap ValidationSupplementary Material 1. Robson Classification of model.

## Data Availability

The datasets used or analysed in the current study are available from the corresponding author upon reasonable request.

## Abbreviations

aHR: Adjusted hazard ratio
BMI: Body mass index
CI: Confidence interval
HIRA: Health Insurance Review & Assessment Service (Korea)
ICD-10: International Classification of Diseases, 10th Revision
IPTB: Iatrogenic preterm birth
IRB: Institutional Review Board
IVF: In vitro fertilization
KM: Kaplan–Meier
NHIS: National Health Insurance Service (Korea)
OECD: Organization for Economic Co-operation and Development
PROM: Premature rupture of membranes
PTB: Preterm birth
SPTB: Spontaneous preterm birth
VIF: Variance inflation factor
WHO: World Health Organization
