# Supplementary material for: Association between maternal risk factors and preterm birth in South Korea: a nationwide cohort study of 795,715 pregnancies
Source: BMC Pregnancy Childbirth. 2026 Feb 10;26:282. doi: 10.1186/s12884-026-08791-1 (PMC12990608; doi:10.1186/s12884-026-08791-1)
Supplement: Supplementary file 1 — Supplementary Material 1. Robinson Classification of the model. Supplementary Figure 1. Kaplan–Meier curves of preterm births according to maternal and clinical factors. Supplementary Figure 2. DAG(Directed Acyclic Graph) Diagram. Supplementary Table 1. General characteristics of factors. Supplementary Table 2. Variable Selections according to comprehensive literature review on related factors. Supplementary File 1. Factors considered in this study (in Detail). Supplementary File 2. Factors According to timeline. Supplementary File 3. Chi-squared test. Supplementary File 4. Calibration Plot. Supplementary File 5. Bootstrap ValidationSupplementary Material 1. Robson Classification of model. [file 12884_2026_8791_MOESM1_ESM.zip › Supplementary Material.docx]

| **Robson Classification** | |
| --- | --- |
| Group 1 | Nullparous women with a single cephalic pregnancy, >=37 weeks gestation in spontaneous labour |
| Group 2 | Nulliparous women with a single cephalic pregnancy, |
| Group 3 | Multiparous women without a previous uterine scar, with a single cephalic pregnancy, >= 37weeks gestation in spontaneous labour |
| Group 4 | Multiparous women without a previous uterine scar, with a single cephalic pregnancy, >=37 weeks gestation who either had labour induced or were delivered by c-section before labour |
| Group 5 | All multiparous women with at least one previous uterine scar, with a single cephalic pregnancy, >=37 weeks gestation |
| Group 6 | All nulliparous women with a single breech pregnancy |
| Group 7 | All multiparous women with a single breech pregnancy, including women with previous uterine scars |
| Group 8 | All women with multiple pregnancies, including women with previous uterine scars |
| Group 9 | All women with a single pregnancy with a transverse or oblique lie, including women with previous uterine scars |
| Group 10 | All women with a single cephalic pregnancy <37 weeks gestation, including women with previous scars |

| Core Variables | Robson Classification | Mapping: Our model included |
| --- | --- | --- |
| Parity | Y/N | Y |
| Previous CS | Y/N | Y |
| Onset of labour | Y/N | Y |
| Number of fetuses | Y/N | Y |
| Gestational age | Y/N | Y |
| Fetal lie and presentation | Y/N | **N (Not available)** |
