# Supplementary material for: Association between maternal risk factors and preterm birth in South Korea: a nationwide cohort study of 795,715 pregnancies
Source: BMC Pregnancy Childbirth. 2026 Feb 10;26:282. doi: 10.1186/s12884-026-08791-1 (PMC12990608; doi:10.1186/s12884-026-08791-1)
Supplement: Supplementary file 1 — Supplementary Material 1. Robinson Classification of the model. Supplementary Figure 1. Kaplan–Meier curves of preterm births according to maternal and clinical factors. Supplementary Figure 2. DAG(Directed Acyclic Graph) Diagram. Supplementary Table 1. General characteristics of factors. Supplementary Table 2. Variable Selections according to comprehensive literature review on related factors. Supplementary File 1. Factors considered in this study (in Detail). Supplementary File 2. Factors According to timeline. Supplementary File 3. Chi-squared test. Supplementary File 4. Calibration Plot. Supplementary File 5. Bootstrap ValidationSupplementary Material 1. Robson Classification of model. [file 12884_2026_8791_MOESM1_ESM.zip › Supplementary Table 1.docx]

| **Factors** | **Condition (ICD-10 Code)** |
| --- | --- |
| Insurance type | National Health Insurance/Medical Aid Recipient |
| Parity | Nulliparous/Multiparous |
| Adolescent Pregnancy | Maternal age <20 |
| History of miscarriage | O00-O08, within 3 years prior to study period |
| False Labor | O47 |
| Severe complications | O13, O14, O15, O36.5, O40, O41, O42, O44, O45 |
| History of preterm birth | O60, within 10 years prior to the study period |
| Artificial reproduction technique  (depends on *in vivo* or *in vitro* fertilization(IVF)) | R6430, R6431, R6432, R6440, R6441, R6442, R6450, R6451, R6452, R6460,  R6461, R6462, R6420, R6491, R6492,  R6493, R6494, R6496, R6481, R6482,  R6501, R6502, R6560  R6471, R6472, R6511, R6512, R6513,  R6514, R6521, R6522, R6523, R6524,  R6510, R6530, R6531, R6540, R6550,  R6532, R6533 |
