# Supplementary material for: Association between maternal risk factors and preterm birth in South Korea: a nationwide cohort study of 795,715 pregnancies
Source: BMC Pregnancy Childbirth. 2026 Feb 10;26:282. doi: 10.1186/s12884-026-08791-1 (PMC12990608; doi:10.1186/s12884-026-08791-1)
Supplement: Supplementary file 1 — Supplementary Material 1. Robinson Classification of the model. Supplementary Figure 1. Kaplan–Meier curves of preterm births according to maternal and clinical factors. Supplementary Figure 2. DAG(Directed Acyclic Graph) Diagram. Supplementary Table 1. General characteristics of factors. Supplementary Table 2. Variable Selections according to comprehensive literature review on related factors. Supplementary File 1. Factors considered in this study (in Detail). Supplementary File 2. Factors According to timeline. Supplementary File 3. Chi-squared test. Supplementary File 4. Calibration Plot. Supplementary File 5. Bootstrap ValidationSupplementary Material 1. Robson Classification of model. [file 12884_2026_8791_MOESM1_ESM.zip › Supplementary Table 2.docx]

Supplementary Table 2.Variable Selections according to comprehensive literature review on related factors.

| **Factors** | **Literature** | | **Reason** | |
| --- | --- | --- | --- | --- |
| **Maternal Age** | ACOG PB 222 (2020); Cleary-Goldman JAMA 2005 | | Advanced age is associated with medical comorbidities (HTN, DM), placental dysfunction, ↑ indicated PTB | |
| **Pregnancy at adolescent** | Chen XK, BJOG 2007; WHO 2018 | | Biological immaturity and socioeconomic disadvantage; ↑ risk of sPTB, LBW. | |
| **Insurance Type** | Kim D, BMJ Open 2023; Blumenshine S, SSM 2010 | | Insurance status is a proxy for socioeconomic status; disadvantaged groups show ↑ PTB, complications. | |
| **History of Stillbirth** | Lamont K, Lancet 2011; Black M, Obstet Gynecol 2008 | | Reflects uteroplacental dysfunction or maternal comorbidities; ↑ recurrence risk of adverse pregnancy including PTB. | |
| **History of Miscarriage** | Oliver A, Hum Reprod Update 2008; McPherson JA, BJOG 2016 | | Recurrent miscarriage suggests cervical/uterine abnormalities; ↑ PTB in subsequent pregnancies. Due to the statistical reason, this factor has been excluded. | |
| **History of Preterm birth** | ACOG PB 234 (2021); Goldenberg RL, NEJM 2008 | | Strongest risk factor; recurrence risk OR 2–3. | |
| **Primiparity** | Shah PS, BMC Pregnancy Childbirth 2011; Blencowe H, Lancet 2012 | | Nulliparas have ↑ obstetric complications, PE, labor dystocia; some studies show modest ↑ PTB. Due to the statistical reason, this factor has been excluded. | |
| **Cervix Insufficiency** | SMFM Consult #70 (2024); Hassan S, NEJM 2011 | | Biomarker for sPTB; validated predictor; effective target for interventions (progesterone, cerclage). | |
| **Recurrent Spontanueous Abortion** | Practice Committee, ASRM 2020; Coomarasamy A, Lancet 2015 | | Associated with uterine anomalies, cervical insufficiency, thrombophilia; ↑ PTB risk in subsequent viable pregnancy. | |
| **Threatened preterm labor** | Clark SL, AJOG 2009; Tita AT, NEJM 2009 | | Uterine contractions and cervical changes that increase the risk of preterm delivery: important modifiable factor. | |
| **Severe complications** | Villar J, Lancet 2006; Ananth CV, Epidemiology 2017 | | Necessitate indicated PTB for maternal/fetal safety; major driver of early preterm births. | |
| **Cervical cerclage** | Sooyoung Oh et al | | Meta-analysis of 71 cohorts: any local cervical treatment increases risks of overall/severe/very early prematurity; risk rises with cone depth (≈2× for >10 mm, ≈3× for >15–17 mm, ≈5× for >20 mm). Multiple conizations raise risk ~4× vs no treatment. | |
| **Endometriosis** | Sooyoung Oh et al | | Systematic review/meta-analysis (39 studies) reports higher risks across outcomes; for preterm birth, pooled OR ≈1.46 (95% CI 1.26–1.69), with the signal persisting in spontaneous conception sub-analyses (heterogeneity high). Mechanisms proposed: placental dysfunction, inflammatory milieu. | |
| **Uterus malformation** | Sooyoung Oh et al | | 2024 systematic review/meta-analysis (32 studies): CUA significantly increases preterm birth (adjusted OR ≈4.34; 95% CI 3.59–5.21), plus PROM, malpresentation, and perinatal loss; risk profiles differ by anomaly type. | |
| **ART(IUI)** | Pinborg A, Hum Reprod 2013; Salmeri N, Hum Reprod Update 2024 | | ART associated with ↑ PTB even in singletons, possibly due to parental subfertility, procedures, or epigenetics. | |
| **(ART)** |  |  |  |  |
| **Factors** | | Literature | | Reason |
| **Income Level** | | Blumenshine S, Soc Sci Med 2010; Braveman P, Annu Rev Public Health 2011 | | Proxy for socioeconomic status. Low income. Due to limited access, decided to be excluded. |
| **Smoking** | | U.S. Surgeon General Report 2014; Stock SJ, BMJ 2012 | | modifiable factor for pregnancy. Due to limited access, decided to be excluded. |
| **BMI** | | Cnattingius S, JAMA 2013; McDonald SD, BMC Pregnancy Childbirth 2010 | | modifiable factor for pregnancy. Due to limited access, decided to be excluded. |
| **Familial Aggregation of preterm birth** | | Clausson B, NEJM 2000; York TP, Am J Obstet Gynecol 2010 | | Shared genetic and environmental factors increase the risk of PTB recurrence. Independent risk factors in multiple cohort studies. Due to limited access, decided to be excluded. |
| **Geographical Diffences** | | Tita AT, NEJM 2009, Hassan S, NEJM 2011 | | The access level of hospital is important. Due to its characteristics, decided to be excluded. |
| **Hospital size** | | - | | This partially demonstrates the level of healthcare services. Due to its statistical instability, decided to be excluded. |
