# Supplementary material for: Association between maternal risk factors and preterm birth in South Korea: a nationwide cohort study of 795,715 pregnancies
Source: BMC Pregnancy Childbirth. 2026 Feb 10;26:282. doi: 10.1186/s12884-026-08791-1 (PMC12990608; doi:10.1186/s12884-026-08791-1)
Supplement: Supplementary file 1 — Supplementary Material 1. Robinson Classification of the model. Supplementary Figure 1. Kaplan–Meier curves of preterm births according to maternal and clinical factors. Supplementary Figure 2. DAG(Directed Acyclic Graph) Diagram. Supplementary Table 1. General characteristics of factors. Supplementary Table 2. Variable Selections according to comprehensive literature review on related factors. Supplementary File 1. Factors considered in this study (in Detail). Supplementary File 2. Factors According to timeline. Supplementary File 3. Chi-squared test. Supplementary File 4. Calibration Plot. Supplementary File 5. Bootstrap ValidationSupplementary Material 1. Robson Classification of model. [file 12884_2026_8791_MOESM1_ESM.zip › Supplementary File 1.docx]

- **Maternal demographics:** Age at delivery (in years), categorised (e.g., <20, 20–29, 30–34, 35–39, ≥40) for descriptive purposes and treated as continuous or categorical in modelling as appropriate; nationality (Korean or foreign-born, as the National Health Insurance Service (NHIS) includes foreigners residing in Korea; included because of possible differences in access or risk); insurance eligibility type (employee insured vs. self-employed/dependent, as a proxy for socioeconomic status).
- **Reproductive history:** Parity (nulliparous vs. multiparous) and any history of PTB before the study period. We determined prior PTB by searching the records for deliveries of each mother before 2013, which were coded as preterm, or any indication of PTB in her medical history (e.g. diagnostic code for previous preterm labour). Given the 10-year window, most prior births of older multiparas were captured if they occurred within or after 2002. A binary variable for history of PTB (yes/no) was created.
- **Assisted Reproduction Technology (*in vitro* fertilisation [IVF]):** Pregnancies conceived through ART were identified using insurance claims for IVF-related procedures (e.g., embryo transfer codes) recorded the year prior to delivery. Since the mid-2010s, many IVF procedures have been reimbursed by the National Health Insurance Service (NHIS), allowing the reliable capture of ART utilisation in administrative claims data. A binary variable (yes/no) was created to indicate conception through ART. Although IVF was strongly correlated with multiple gestations, we retained ART as an independent covariate to reflect potential additional risks beyond multiplicity such as underlying subfertility or maternal factors associated with IVF.
- ART was not treated as the primary exposure of interest, but rather as one of several maternal and healthcare-related predictors included in the multivariable model, given its established association with PTB and its rising prevalence in South Korea. To enhance clarity, Table 1 presents ART as a single binary variable and the full list of procedure codes used to define ART is provided in Supplementary Table S1 to ensure transparency and reproducibility.
- **Maternal comorbidities:** We included key pre-pregnancy and pregnancy-acquired health conditions that influence PTB risk (16, 17). Outpatient and inpatient records were obtained for each patient. Severe pregnancy complications were incorporated into the model because they represent common obstetric pathways leading to preterm delivery. For example, preeclampsia and gestational hypertension can necessitate early delivery due to the risk of maternal seizures, stroke, or placental insufficiency. Placenta previa and placental abruption frequently result in antepartum haemorrhage and often require urgent delivery to prevent maternal and foetal death. Premature rupture of membranes (PROM) exposes the foetus to infection and cord prolapse, which may precipitate preterm labour or necessitate delivery. These conditions are not “causes” in the sense of primary exposures but rather proximal determinants that obstetricians routinely manage to balance maternal and neonatal survival. For a general epidemiological audience, their inclusion in the model should be interpreted as risk indicators that improve predictive accuracy rather than upstream social or biological exposures (Supplementary Table 2).
- **False Labor:** This factor was included as a covariate because it represents the clinical episodes of uterine contractions and cervical changes that often precede true PTB. Although not all cases progress to delivery, the condition reflects heightened obstetric risk and maternal healthcare utilisation and thus, may serve as an important predictor of PTB in risk stratification models. Incorporating False Laborallowed the model to capture a clinically relevant intermediate stage between normal pregnancy and imminent preterm delivery, thereby improving both predictive accuracy and epidemiological interpretation.

***Outcome***

**Preterm Birth (ICD code: O60.1):** PTB was classified into spontaneous and iatrogenic PTB (IPTB) based on the ICD-10 diagnostic and procedure codes. Spontaneous PTB (SPTB) was defined as preterm labour or premature rupture of membranes leading to delivery, identified using the ICD-10 code O60.1 (preterm spontaneous labour with preterm delivery or preterm labour without delivery, unspecified). These codes capture PTBs resulting from the natural onset of labour or obstetric complications. IPTB was defined as provider-initiated early delivery before 37 weeks of gestation, typically via induction of labour or caesarean section for maternal or foetal indications (e.g. severe preeclampsia, foetal growth restriction, placental abruption) and was identified using delivery procedure codes and ICD-10 code O60.3. To ensure accurate classification, the cohort was restricted to live singleton deliveries. Cases involving macrosomia (ICD-10 O36.6, or birth weight >4,000 g) were excluded, as they do not represent typical PTB scenarios and may have biased the associations. The primary outcome was time to PTB, which was defined as delivery before 37 weeks of gestation. Gestational age, measured in completed weeks from the last menstrual period or the estimated conception date (as recorded in the claims data), served as the underlying timescale. Pregnancies reaching full term (37–42 weeks) or beyond were considered censored at the time of delivery. Several variables were excluded from the final model due to data limitations, clinical considerations, or methodological concerns. Work status and household income were deemed unreliable or unavailable and therefore could not be incorporated. Multiple gestations were excluded based on clinical guidance, because births occurring after 35 weeks in multiple pregnancies may not be appropriately classified as preterm, thereby reducing the potential for misclassification.

- - Gestational hypertension with significant proteinuria (preeclampsia) (O14)
  - Gestational pregnancy-induced hypertension without significant proteinuria (O13)
  - Eclampsia (O15)
  - Polyhydramnios (O40)
  - Other disorders of amniotic fluid and membranes (O41)
  - Premature rupture of membranes (O42)
  - Placenta previa (O44)
  - Premature separation of placenta (placental abruption)
  - Antepartum haemorrhage, not elsewhere classified (O46)
  - Diabetes mellitus arising during pregnancy, unspecified (gestational diabetes, type not specified) (O244)
  - Maternal care for known or suspected poor foetal growth (O365)
  - Recurrent spontaneous abortion (O262)
  - Endometriosis (N80)

These conditions were primarily assessed as binary indicators (present or absent) during pregnancy. For chronic conditions, a diagnosis was based on the history or pregnancy of the woman.
