# Supplementary material for: Association between maternal risk factors and preterm birth in South Korea: a nationwide cohort study of 795,715 pregnancies
Source: BMC Pregnancy Childbirth. 2026 Feb 10;26:282. doi: 10.1186/s12884-026-08791-1 (PMC12990608; doi:10.1186/s12884-026-08791-1)
Supplement: Supplementary file 1 — Supplementary Material 1. Robinson Classification of the model. Supplementary Figure 1. Kaplan–Meier curves of preterm births according to maternal and clinical factors. Supplementary Figure 2. DAG(Directed Acyclic Graph) Diagram. Supplementary Table 1. General characteristics of factors. Supplementary Table 2. Variable Selections according to comprehensive literature review on related factors. Supplementary File 1. Factors considered in this study (in Detail). Supplementary File 2. Factors According to timeline. Supplementary File 3. Chi-squared test. Supplementary File 4. Calibration Plot. Supplementary File 5. Bootstrap ValidationSupplementary Material 1. Robson Classification of model. [file 12884_2026_8791_MOESM1_ESM.zip › Supplementary File 3.docx]

| **Variable** | **PTB = Yes, n (%)** | **PTB = No, n (%)** | **Total** | **p-value** |
| --- | --- | --- | --- | --- |
| **Age group** |  |  |  | <0.001 |
| 20–29 | 20,230 (9.3) | 197,200 (90.7) | 217,430 |  |
| 30–39 | 42,760 (8.0) | 491,760 (92.0) | 534,520 |  |
| 40–49 | 3,520 (8.3) | 38,245 (91.7) | 41,765 |  |
| **Insurance type** |  |  |  | <0.001 |
| Medical Aid | 440 (10.9) | 3,600 (89.1) | 4,040 |  |
| National Health Insurance | 54,460 (6.9) | 736,215 (93.1) | 790,675 |  |
| **History of preterm birth** |  |  |  | <0.001 |
| Yes | 4,565 (78.6) | 1,240 (21.4) | 5,805 |  |
| No | 50,335 (6.4) | 739,575 (93.6) | 789,910 |  |
| **False labor** |  |  |  | <0.001 |
| Yes | 50,700 (10.3) | 441,600 (89.7) | 492,300 |  |
| No | 4,200 (1.3) | 299,215 (98.7) | 303,415 |  |
| **Severe complications** |  |  |  | <0.001 |
| Yes | 38,100 (16.4) | 194,200 (83.6) | 232,300 |  |
| No | 16,800 (3.0) | 546,615 (97.0) | 563,415 |  |
| **Primiparity** |  |  |  | <0.001 |
| Yes | 13,900 (7.7) | 167,000 (92.3) | 180,900 |  |
| No | 41,000 (6.7) | 573,815 (93.3) | 614,815 |  |
